# Stress convulsions in rodents: within a weight-of-evidence framework for human seizure risk

**DOI:** 10.3389/ftox.2025.1600816

**Published:** 2025-07-17

**Authors:** Joseph J. DeGeorge, Monica R. Metea

**Affiliations:** ^1^ Bianca Holdings, LLC, Waymart, PA, United States; ^2^ Preclinical Electrophysiology Consulting, LLC, Boston, MA, United States; ^3^ Lucerum, Inc, Boston, MA, United States

**Keywords:** stress-induced convulsions, psychogenic nonepileptic seizures (PNES), electrographic seizures (ES), rodent models in regulatory toxicology, preclinical seizure liability assessment, weight-of-evidence (WoE) framework, environmental stressors in regulatory testing, regulatory decision-making in CNS safety

## Abstract

In research settings, rodents exhibit a well-documented sensitivity to stress-induced behavioral alterations ranging from stereotypy to convulsions. These events complicate preclinical drug safety assessments where establishing a No-Observed-Effect Level (NOEL) requires distinguishing true pharmacologic seizures from stress-related convulsions, including a type lacking electrographic cortical correlates, referred to as psychogenic nonepileptic seizures (PNES). Stress triggers in preclinical settings include environmental factors and systemic conditioning effects of investigational drugs unrelated to seizure risk. Stress-induced behaviors can bias safety assessments by creating false-positive findings of seizure liability incorrectly attributed to the test compound. This paper highlights situations when stress conditioning is present during rodent seizure liability studies and proposes a Weight-of-Evidence (WoE) approach to differentiate between drug-induced ES and stress-conditioned PNES. It supports applying context-specific criteria for regulatory considerations especially when convulsions are absent in higher species, when there are inconsistent findings across facilities, and when rodents present stereotypy and lack of neuropathological evidence of drug-induced seizures. This approach aims to minimize the misinterpretation of stress-related artifacts as true pharmacologic seizures, providing a framework for more reliable and translatable seizure liability assessments.

## 1 Introduction

Rodents are commonly used in preclinical safety assessments to establish a No-Observed-Effect Level (NOEL) for seizure liability. However, their susceptibility to stress-induced convulsions complicates interpretation, particularly when distinguishing pharmacologically-induced seizures from behavioral artifacts such as stereotypy and from psychogenic nonepileptic seizures (PNES) which, unlike epileptiform electrographic seizures (ES), are conditioned responses lacking cortical paroxysms which can occur independent of a compound’s pharmacologic action.

This paper addresses the challenges associated with interpreting convulsions in rodents observed during routine regulatory general toxicology studies, where animals are not seizure models, i.e., convulsions are not intentionally experimentally induced or expected and observations are based on behavioral signs without EEG confirmation. These limitations underscore the need for a structured framework to differentiate between true pharmacologic seizure risk and stress-related phenomena. We summarize mechanisms underlying both stress conditioned PNES and ES and outline heuristics differentiating between them in rat while recognizing that they may not be always feasible for routine toxicology studies. In these situations, we advocate for a WoE approach to the program data in the regulatory context. Developed from a foundation of peer-reviewed literature and regulatory precedent, this manuscript presents a framework to inform scientific and policy discussions and is targeted to preclinical drug development professionals.

## 2 Mechanisms of stress-induced convulsions in rodents

While stress-indiced convulsions are classically associated with genetically predisposed strains (Skradski et al., 1998), they have also been well-documented in non-epileptic, wild-type rodents following induced acute environmental stress such as sound exposure. We reference this paradigm not to suggest a purely auditory mechanism for stress convulsions, but to illustrate that intense environmental stimuli can induce convulsive responses even in neurologically intact animals without chemically-induced sensitization, highlighting the species’ intrinsic sensitivity to stress-related seizures. In the context of regulatory toxicology studies, where environmental variables such as facility noise, room entry, handling, and procedural timing are not systematically controlled, similar stress-induced convulsions can emerge unpredictably even in otherwise well operated facilities. These responses, while not induced intentionally, may manifest as spontaneous convulsive events and confound interpretation of drug safety profiles.

“Audiogenic seizures,” i.e., exposure to sound triggering convulsions, are an experimental stress research paradigm established over more than 7 decades (Griffiths, 1962; Ross and Coleman, 2000; Seyfried, 1979; Eells et al., 2004), demonstrating that environmental factors can alter even normal phenotypes towards convulsion. Audiogenic convulsions have been observed in genetically predisposed strains (Todorova et al., 1999), kindled rodents, and notably, in wild-type rodents that are not inherently seizure-prone, occurring in response to stressors in the absence of any pharmacologic intervention (Ross and Coleman, 2000; Griffiths, 1962; Heinrichs, 2010; Poletaeva et al., 2017).

Beyond audiogenic convulsion example, the fact that both seizure-susceptible and non-susceptible rodent models routinely develop convulsive episodes following exposure to stressors, underscores the intrinsic sensitivity of the species to stress-related convulsions. In this context it is important to recognize that the term “seizure” usually refers to a systemic modulation of intrinsic synchrony resulting from reduced inhibition of subcortical or limbic drive to the cortex, rather than cortical neurotoxicity.

Three pathways relevant to rodents have been implicated in stress-induced seizures. These mechanisms are not intended as an exhaustive account, but are sufficient to support the biological plausibility of spontaneous stress-related convulsions in regulatory study settings.

(1) *Hippocampal activation:* Stressor exposure drives hippocampal electrophysiological activation (Buwalda et al., 2005) and plasticity changes (McEwen and Magarinos, 2001) involving a stress circuit composed of “resonator” neurons. These neurons are sensitive to perceived noxious stimuli by responding to otherwise normal input with an abnormal discharge that often triggers a seizure (Eggers, 2007), suggesting that the tonic inhibitory functions of the hippocampus are modulated by stress-induced excitation (Drage et al., 2002).
(2) *Noradrenergic neurotransmission:* Primarily mediated by the locus coeruleus (LC), which provides the major arousal-related (Aston-Jones and Bloom, 1981) adrenergic input to many brain regions including the cerebral cortex. Evidence suggests that increased LC activity and norepinephrine (NE) release act as a protective mechanism against seizures, and disruptions in this system lead to heightened seizure susceptibility and even *status epilepticus* (Weiss et al., 1990; Giorgi et al., 2003).
(3) *Hypothalamic-pituitary-adrenal (HPA) axis*: Stressor exposure activates the adrenocortical hormone system, with seizure severity correlated to the sensitivity of the system to the excitatory effects of glucocorticoids (Heinrichs, 2010). Compelling evidence suggests that stressors capable of triggering seizures also elevate glucocorticoid levels. Activation of the hypothalamic-pituitary-adrenal (HPA) axis increases glucocorticoid levels (e.g., *corticosterone* in rodents), which in turn lower the seizure threshold (Maguire and Salpekar, 2013). For instance, exposure to intense noise commonly used to provoke audiogenic seizures initiates a neuroendocrine stress cascade, including activation of the HPA axis (Michaud et al., 2003), and handling-induced convulsions in rodents produce significant elevations in plasma corticosterone (Balcombe et al., 2004). The neural circuits which mediate the pro-convulsant actions of glucocorticoids may also include stress neuropeptide circuits which initiate HPA activation within the brain (Baram and Hatalski, 1998), and other related mechanisms.

Glucocorticoid-induced activation of brain activity provides a plausible mechanism for both short-term neural and behavioral arousal as well as long-term escalation of stress-related processes. Shortly after initial exposure to stress (i.e., priming), glucocorticoid hormones increase cellular excitability in the hippocampus (Joëls, 2008), resulting in encoding of stress-related information (de Kloet et al., 2008), which enhances intrinsic spike-wave discharges (Schridde and van Luijtelaar, 2004). This can facilitate both conditioned convulsions (unrelated to a drug) (Roberts et al., 1994), as well as lower the seizure threshold for ES via kindling, if an administered drug is indeed seizurogenic yet at a much higher dose level (Karst et al., 1999). Further, chronic stress post the sensitization phase can establish continuation of convulsive activity (Joëls et al., 2007), falsely presenting as a chronic seizurogenic drug effect. In short, acute stress responses rapidly enhance hippocampal excitability, facilitating seizure onset, while chronic stress leads to neuroplastic changes, potentially leading to persistent or recurrent epileptogenesis (Joëls et al., 2007).

Short-term stress-induced HPA priming and long-term neuroplasticity (de Kloet et al., 2008) can also participate in a conditioned responses to a drug that poses a strong systemic strain, yet lacking proconvulsant activity. In such instance, acute environmental priming factors (facility cues) may trigger stereotypy to systemic drug-induced stress (e.g., gastric, etc.) in a manner correlated to the strength of the prediction.

Further, compared to primates, rodents exhibit distinctions in brain anatomy. The primate neocortex is highly gyrencephalic with expanded associative areas, increased inhibition and greater intracortical connectivity, whereas rodents have a lissencephalic cortex with laminar organization and fewer long-range inhibitory interneurons (Elston et al., 2011; Wynshaw-Boris et al., 2010), associated with a higher baseline excitability (Traub et al., 2005; Metea et al., 2015; Uylings et al., 2003). This difference has implications for subcortical seizure onset and propagation. Additionally, glutamate and GABAergic neurotransmission, both key players in seizure dynamics, exhibit species-specific differences (Mendu et al., 2012; Bekenstein and Lothman, 1991; Semple et al., 2013). The rodent brain exhibits different GABAergic interneurons with fewer inhibitory double bouquet cells, which are known to exert strong feedforward inhibition in primates (DeFelipe, 1999).

Collectively, mechanistic and anatomical considerations suggest that stress-exposed rats are at an increased risk of conditioned seizures from multifactorial causes.

## 3 Psychogenic nonepileptic seizures (PNES): clinical and preclinical parallels

### 3.1 PNES vs. epilepsy in clinical practice

While stress-induced seizures can spread as cortical paroxysms, not all convulsions triggered by stress are associated with cortical activation. A subset of these events may instead fall under the category of *psychogenic nonepileptic seizures (PNES)*.

Clinically, PNES (or Functional Neurological Disorder - FND) are a condition at the intersection of neurology and psychiatry, presenting as convulsive events associated with heightened emotional reactivity, and thought to affect the regulation of motor output (Diez et al., 2021), (Boutros et al., 2019). PNES are considered the manifestation of an acquired network disorder related to emotional learning/reactivity and motor control circuitry involving limbic regions (Amiri et al., 2021; Balachandran et al., 2021). PNES are currently considered a multi-factorial condition involving complex interactions between environmental, genetic, and psychological factors, with experiences of childhood trauma being the most common risk factor (Yang et al., 2023; Goldstein et al., 2019).

PNES lacks ictal epileptiform discharges or seizures on EEG, although the EEG may show other abnormal nonspecific changes; they are resistant to traditional antiseizure drugs; and at least partially responsive to induction or provocative techniques. Although PNES convulsions do not display abnormal cortical synchrony, their repeated presence can trigger secondary paroxysms (sudden, transient bursts of abnormal EEG activity, e.g., seizure events), highlighting the intricate interplay between subcortical stress pathways and seizure manifestation (Benbadis et al., 2001; Dickinson and Looper, 2012). In epilepsy clinics, PNES are detected at a relatively high percentage varying from 10% to over 50% (Benbadis, 2006; Benbadis et al., 2001).

PNES are confirmed by simultaneous video and EEG in a neurology setting, confirming an absence of ictal EEG changes and the presence of normal awake EEG rhythms before, during, and immediately after the event (Benbadis, 2006; LaFrance et al., 2013). Activation procedures are critical for the differential diagnosis of PNES (Benbadis, 2006), and are a common intervention for psychogenic movement disorders (Brown, 2006). No single biomarker successfully differentiates PNES from ES, although current efforts are underway to identify additional clinical chemistry markers differentiating between PNES and epileptiform variants (Sundararajan et al., 2016).

### 3.2 PNES parallels in rodents in the context of general toxicology studies

Reports from Contract Research Organizations (CROs) conducting routine toxicology studies (Gauvin et al., 2018; Satomoto et al., 2012) where seizures are recorded based on behavioral observation alone, without EEG or detailed Racine scale staging (Racine, 1972) confirm the presence of conditioned stress-induced convulsions with PNES features in rodents, elicited ususally by reactivity to handling, fearful situations, unexpected light changes, or sound. These conditions are commonly associated with dosing or room observations.

Stress convulsions in rats share features with PNES in humans, i.e., acquired sensitivity to environmental cues paralleling trauma, and a strong association with triggers and autonomic dysregulation. The presence of overlapping non-convulsive behaviors such as stereotypy, conditioned responses to cues, and the absence of postictal suppression usually allows for a functional comparison. Given these shared features, PNES terminology can be applied to rodents to describe stress-induced conditions representing maladaptive motor phenomena (Gauvin et al., 2018).

### 3.3 Continuum of stereotypy and PNES in rodents

Rat stereotypy and PNES variants are on the same continuum of behaviors sharing mechanistic pathways. Stereotypy in rats is a well-documented non-seizurogenic phenomenon, with invariant behaviors such as excessive grooming, jumping, circling, route-tracing, biting, pacing, shaking, twitching, paddling, etc., often observed in captive environments (Garner, 2005). Rodent stereotypy measurement has a long history in the field of psychopharmacology (Kelley, 2001), first used to describe the physiological effect of stimulant or excitatory drugs like apomorphine or amphetamine, yet spontaneous stereotypy was also documented as a result of stress (Powell et al., 1999).

Mechanistically, stereotypy is believed to involve dopaminergic dysregulation within the basal ganglia and the nigrostriatal or mesolimbic pathways responsible for motor control and reinforcement learning (Powell et al., 1999). Additionally, glutamatergic and GABAergic imbalances contribute to the persistence of these behaviors by impairing inhibitory control over motor circuits. In rodents, stereotypy is often linked to dopaminergic dysregulation within the striatum, where excessive activation leads to rigid, compulsive motor patterns (Canales and Graybiel, 2000), and exacerbated by chronic stress exposure (Garner, 2005).

Stereotyped non-epileptiform hyperkinesis presents rhythmic side-to-side swings of the head and torso and pronounced pendulum-like movements, abortive or repetitive movements, or stereotyped jumps or shakes (Kelley, 2001; Powell et al., 1999; Garner, 2005). The alternating nature of the movement during stereotypies often appears sufficiently similar to clonic–tonic convulsions where rhythmicity of movements is elicited in alternations between muscle contractions and relaxations. To complicate matters further, stereotypy can occasionally be present as an intermediate phase when a shift from a catatonic to an epileptiform seizure occurs (Ryazanova et al., 2023), and the association of these events in sequence may add to the difficulty of distinguishing between them.

In general, stereotypies are pathological motor responses to environmental factors involving dysregulation of basal ganglia, limbic system, or the prefrontal cortex, which regulate movement, emotional responses, and behavioral flexibility. These phenomena can be viewed through the lens of learned or conditioned responses, developing when an animal repeatedly engages in a behavior that provides a temporary coping mechanism and reinforced even when it is no longer adaptive.

While stereotypies/PNES and drug-induced seizures arise from distinct neural mechanisms, they exhibit sufficiently similar behavioral features and clinical observations. However, since stereotypy can manifest with or without underlying epileptiform activity, it is not a reliable prodromal indicator of seizure when stress conditioning is suspected.

### 3.4 Environmental stressors in the course of toxicology studies

Preclinical studies introduce multiple environmental stressors that can function as triggers/cues for conditioned responses and can vary between facilities.

Dosing is a stress factor and can induce anticipatory stress responses, particularly when paired with aversive handling techniques, or with restraint (Balcombe et al., 2004). Routine laboratory noise, across humanly audible range or ultrasonic, can also act as an unexpected stressor (Turner et al., 2005). Because rodents are nocturnal, facility activities occurring during the day are inherently more stressful. Many of these factors resemble cued fear conditioning paradigms (Buccafusco, 2009), variants of the stress response models which can alter the natural freezing response to both contextual and auditory conditional stimuli and participate indirectly to disinhibition of repetitive movements (Goosens and Maren, 2001).

Variations in social housing, bedding material, and cage type can also impact stress resilience (Van Loo et al., 2003). Rodents are highly social animals, and isolation has been shown to increase anxiety behaviors and dysregulate stress hormone levels. Social buffering (Kiyokawa et al., 2004), where the presence of cage mates helps dampen stress responses, is a well-documented phenomenon that can reduce the likelihood of stress conditioning.

Overall, the elements of a conditioning context require features bound into a conjunctive representation, sharing mechanisms common to formation of memories (Rudy et al., 2004). Dissociating learning due to the cue and the context adversity is difficult, as in some cases the stress related to the cue itself may also interfere with contextual learning (Buccafusco, 2009).

Inter-facility differences in husbandry, environmental enrichment, and personnel training can significantly affect stress exposure (Gauvin et al., 2018). Therefore, when variability in the incidence of convulsions across facilities is observed, it may indicate underlying stress conditioning.

### 3.5 Differentiating PNES from drug-induced seizures in toxicology studies

In regulatory general toxicology studies, behavioral seizures are typically described in general terms such as “clonic,” “tonic-clonic,” or “convulsion,” often without further phenotypic classification. No mandatory standardized behavioral scoring system is routinely applied, (although general parameters of duration may be included), and observational criteria may vary across facilities depending on local procedures. The Racine scale widely cited in epilepsy research and developed for induced seizure models in preconditioned animals is not applied to neurologically normal animals in toxicology settings. This lack of resolution of general toxicology study convulsion observations contributes to the ambiguity of convulsive findings.

Since stress-induced convulsions and drug-induced seizures may present with similar motor manifestations, their differentiation requires a systematic approach appropriate for toxicology settings as follows:

(1) *EEG:* The defining characteristic of electrographic seizures is a clear progression of EEG with preictal, ictal, and postictal phases (Metea et al., 2015), (Schomer and Silva, 2018). In contrast, during PNES the EEG may be abnormal but will lack ictal EEG, and no postictal abnormalities will be present (Izadyar et al., 2018). Recovery from postictal attenuation in true seizures is slow (minutes) due to neuronal exhaustion, whereas PNES events show rapid EEG normalization.
(2) *Postictal behaviors:* True seizures often result in a period of neurological impairment, whereas PNES episodes typically do not. A very fast recovery after a convulsion suggests PNES (Leibetseder et al., 2020).
(3) *Prodromal behavioral signs: *True seizure usually displays prodromal behavioral signs immediately prior to the event, while PNES episodes may appear abruptly without prior motor abnormalities. Note that since stereotypy can manifest with or without underlying epileptiform activity, it is not a reliable prodroma of drug-induced seizure.
(4) *Identifying the environmental features of a conditioned response:* If convulsions appear consistently after handling, dosing, or technician entry into the room, a learned stress response should be suspected. A drug-induced epileptiform seizure will occur even in the absence of stressors when the target is engaged (usually C_max_ – related). PNES episodes can be induced by noise or stress outside of C_max_.
(5) *Distraction and reflex responses:* Behavioral responsiveness and reflex testing are key differentiating factors similar to the induction techniques used for identifying clinical psychogenic seizures. ES impair awareness and responsiveness, while PNES events often allow partial or full preservation of voluntary responses. During an PNES episode, a simple auditory or tactile reflex-inducting stimulus (e.g., loud clicker, tail pinch, etc.) can be applied to assess motor and sensory reactivity (Gauvin et al., 2018). Note that voluntary movements can be impaired by fear-related “freezing” (Buccafusco, 2009), therefore two or more techniques should be attempted to assess the presence of reflexes.
(6) *Dose dependency:* A dose-dependent increase in seizure frequency or incidence is expected with neurotoxic or proconvulsant compounds (Gauvin et al., 2018). However, a dose-dependent increase in conditioning stimuli strength could lead to a PNES dose relationship. In such cases, careful evaluation of postictal behavior and reflex testing becomes essential.
(7) *Severity: *Convulsions induced by a neurotoxic substance often escalate in incidence and severity over time, whereas psychogenic events remain short and self-terminating without postictal events even when their incidence increases due to generalization of cue context.
(8) *Activation of antagonistic muscle groups:* Generalized seizure-driven convulsions show coordinated movements such as tonic-clonic alternations, while PNES convulsions may lack these features.
(9) *Social contagion:* PNES convulsions may be triggered in response to environmental stressors or observed convulsions in cage mates (Gauvin et al., 2018). However, true seizures in cage mates can also induce stress, therefore social contagion alone is not a sufficient differentiator.

If stress-conditioning is suspected in rat while all other preclinical and clinical data (including EEG, alternate species, or human safety data to date) remain seizure-free (or occurring at high multiples exposure in the severe toxicity range), the rat convulsive events have a high likelihood of being PNES and relying on more predictive species may be justified.

This distinction is particularly important when canine toxicology data show a promising CNS safety profile, as dogs are inherently seizure-sensitive (Berendt et al., 2015; Forsgård et al., 2019; DaSilva et al., 2020). However, the implications of using dogs in seizure liability assessments differ from those of stress-induced PNES in rats. Unlike rats, dogs are susceptible to true idiopathic epilepsy that may be triggered at lower doses of an investigational drug with actual proconvulsant properties.

We present these simple distinguishing criteria specifically because routine toxicology studies are not designed to differentiate seizure subtypes. Animals are normal and not seizure-prone models, electroencephalographic data are rarely collected, and convulsive events are typically documented based on gross behavioral observation. As a result, behaviors consistent with stress-induced stereotypy or PNES may be misclassified as epileptiform seizures. The framework we propose is designed to bring greater structure and interpretive clarity to these ambiguous observations. Importantly, it offers feasible, low-burden strategies such as reflex testing, context evaluation, and timing relative to procedural stressors, that can be readily implemented in ongoing studies to improve confidence in seizure liability assessments.

### 3.6 Balancing feasibility and diagnostic value of rat EEG

From a regulatory and scientific standpoint, EEG remains the definitive tool for confirming electrographic seizure activity, and differentiating PNES from ES. However, in routine toxicology studies, EEG is rarely implemented, and animals are not seizure-prone models. Consequently, events are recorded behaviorally without electrographic validation, and standardized seizure staging systems such as the Racine scale developed for controlled, induced seizure paradigms is not fesible in this context. It is only in the context of highly resource-intensive follow-up studies in rodent that EEG is usually deployed.

Moreover, when stress conditioning is suspected, it is also important to recognize both the value and limitations of EEG measurements in additional focused studies. While EEG is the gold standard for differentiating between ES and PNES, both can be triggered by stress in the absence of a drug effect. EEG does not inherently differentiate whether the observed response is due to a direct drug effect or a conditioned stress response and should be used selectively, when distinguishing between these two mechanisms provide meaningful insight into the interpretation of seizure risk (e.g., when PNES alone is suspected).

It is essential to distinguish these scenarios from the use of EEG as a standard method for assessing drug-induced seizure risk. *When no substantial evidence suggests stress conditioning in rats, EEG remains a crucial tool for identifying drug-induced seizure risk and determining a No Observed Adverse Effect Level (NOAEL)*.

## 4 Discussion

### 4.1 Operational preparedness

Given the impact of convulsions in non-clinical studies and the confounding potential of stress-related conditioning in rodents, there is a need for greater awareness and operational preparedness among facilities conducting rat studies. Significant improvements could be achieved by ensuring test facilities are prepared to detect stress conditioning and differentiate between ES and PNES through distraction techniques, assessing associations with potential triggering cues (e.g., light-dark cycle interruptions, handling prior to dosing), and evaluating their relationship to the maximum observed concentration of a drug in plasma following administration, or C_max_. Late-onset convulsions are not uncommon in chronic rat toxicity studies and may arise from multiple factors, including the test article, age, stress conditioning, or a combination thereof. Even when a definitive conclusion cannot be drawn from a single study, it is recommended that study directors routinely document and contextualize potential stress conditioning effects, providing a more comprehensive and transparent foundation for regulatory discussions.

It is of value to discern stress-related convulsions, from those related to severe systemic toxicity. Kidney toxicity can lead to uremia, metabolic acidosis, or electrolyte imbalances (e.g., hyponatremia, hyperkalemia, hypocalcemia). Gastrointestinal (GI) toxicity can cause dehydration, nutritional deficits, or metabolic imbalances and systemic inflammation, all of which lower the seizure threshold by altering neuronal excitability. These factors should be evaluated in a WoE framework to ensure a more accurate attribution of convulsive risk.

### 4.2 Regulatory framework

For programs under the US FDA/CDER, restrictions on human exposure may be imposed on first-in-human trials (FDA, 2005) and further limitations on maximum clinical doses as outlined in ICH M3 (R2) (ICH M3, 2009) and its Q&A (ICH guideline M3, 2012). This typically leads to additional safety margin restrictions on both AUC and C_max_, often limiting human exposure to 1/10th of the NOAEL for convulsions in the most sensitive species, even in the absence of such events in humans. Other regulatory agencies, such as the MHRA and EMA, may place greater emphasis on clinical experience when human data are available, requesting clinical dose escalation modifications or enhanced monitoring.

When stress-induced events are suspected in rats, we propose adopting a Weight-Of-Evidence (WoE) framework to assess the presence of PNES, considering events in all species, the test agent’s on- and off-target effects relevant to seizure mechanisms, the susceptibility of the clinical trial population, and the fesibility of detecting prodromal activity in humans. Alternative species should be emphasized when multiple indicators of rodent stress conditioning are present. Specifically, if convulsions exhibit PNES features, facility-dependent variability, lack postictal events or pathology findings, and are either absent or occur only at substantially higher exposure multiples in other species, they should be viewed as suspect.

Failing to remove bias introduced by stress into safety assessments can lead to false-positive seizure liability findings that may result in premature termination of otherwise viable drug candidates. Parsing PNES from epileptiform events is essential to ensure that decision-making reflects true translational risk. When the root cause of convulsive events is stress-conditioning rather than pharmacologic neurotoxicity, recognizing this distinction prevents the misallocation of resources, prolonged delays, and unnecessary animal use, while preserving confidence in drug safety. While additional non-clinical investigations may sometimes be necessary, in many cases a targeted proportionate approach can safeguard clinical subjects and address regulatory concerns without excessive delays, resource expenditures, and increased animal use.

We believe that by applying a WoE to convulsion observations, a more appropriate non-clinical and clinical investigative program can be developed without sacrificing safety for clinical subjects and, leading to a more rapid and efficient drug development of clinically important therapeutics.

Table 1 outlines a first attempt towards key considerations that can increase or decrease the relevance of rodent convulsions to human risk assessment.

**TABLE 1:** WoE Criteria for Linking Rodent Convulsions to Stress-Induced Mechanisms.

WoE Category	Low Probability of Stress Cause	High Probability of Stress Cause
Rodent Study-Related		
Timing	Aligned with Test Article^c^ T_max_	Related to dosing or handling
Onset of convulsions	Early after study initiation	Late after study initiation (chronic studies)
Response to provocative intervention during convulsion	No impact on convulsion	Intervention disrupts convulsion
Behavioral correlates	Exhibits pre- and postictal abnormal behaviors	Rapid self-termination of convulsion, full and fast recovery, no prodromal behaviors; Predominantly stereotypy with no progression
CNS-related clinical observations (other than convulsions)	Dose-and T_max_ - related in duration, frequency, severity and persistence^a^	Absent, minor, or transient; Not dose-related
EEG^b^	Preictal epileptiform morphologies and postictal attenuation	No EEG epileptiform abnormalities before, during or after convulsions, and no electrographic seizures
Preclinical Cross-Study Evidence		
Species impacted	Observed both in rodents and non-rodents	Only observed in rat
Studies impacted	Consistent across all rat studies at multiple facilities	Large variance across studies with different environmental conditions
Off-target seizure liability	Convulsions associated with T_max_ of metabolite	Independent of metabolite T_max_
Drug or metabolite accumulation	Substantial accumulation at relevant doses	Minimal accumulation or similar concentrations not associated with convulsion in acute settings
Additional Clinical Considerations		
Existing clinical data	Human data available and not indicative of convulsive risk	
Phase I intended clinical study setting	Inpatient observations with increased neurologic monitoring	
Clinical target/class	Not associated with human risk; Not associated with seizure pathways	
Clinical indication	CNS (with plan for increased neurologic monitoring)	
Clinical study endpoints	Seizure outcome assessed as endpoint	

^a^ May warrant follow-up EEG investigation.

^b^ Not commonly included in general rodent toxicology studies and challenging to add in an ongoing study, unlike in large animals.

^c^ Refers to any compound, substance, or formulation being evaluated in a nonclinical study for its pharmacological, toxicological, or safety profile.

## Data Availability

The original contributions presented in the study are included in the article, further inquiries can be directed to the corresponding author.
